# The genome sequence of the Puss Moth, *Cerura vinula* (Linnaeus, 1758) (Lepidoptera: Notodontidae)

**DOI:** 10.12688/wellcomeopenres.24919.1

**Published:** 2025-10-03

**Authors:** David C. Lees, Natural History Museum Genome Acquisition Lab, Wellcome Sanger Institute Tree of Life Management, Samples and Laboratory team, Wellcome Sanger Institute Scientific Operations: Sequencing Operations, Wellcome Sanger Institute Tree of Life Core Informatics team, Tree of Life Core Informatics collective, Darwin Tree of Life Consortium

**Affiliations:** 1 Natural History Museum, London, England, UK

**Keywords:** Cerura vinula; Puss Moth; genome sequence; chromosomal; Lepidoptera

## Abstract

We present a genome assembly from an individual male *Cerura vinula* (Puss Moth; Arthropoda; Insecta; Lepidoptera; Notodontidae). The assembly contains two haplotypes with total lengths of 690.59 megabases and 688.27 megabases. Most of haplotype 1 (99.72%) is scaffolded into 19 chromosomal pseudomolecules, including the Z sex chromosome. Haplotype 2 was assembled to scaffold level. The mitochondrial genome has also been assembled, with a length of 16.79 kilobases. This assembly was generated as part of the Darwin Tree of Life project, which produces reference genomes for eukaryotic species found in Britain and Ireland.

## Species taxonomy

Eukaryota; Opisthokonta; Metazoa; Eumetazoa; Bilateria; Protostomia; Ecdysozoa; Panarthropoda; Arthropoda; Mandibulata; Pancrustacea; Hexapoda; Insecta; Dicondylia; Pterygota; Neoptera; Endopterygota; Amphiesmenoptera; Lepidoptera; Glossata; Neolepidoptera; Heteroneura; Ditrysia; Obtectomera; Noctuoidea; Notodontidae; Cerurinae; *Cerura*; *Cerura vinula* (Linnaeus, 1758) (NCBI:txid987900)

## Background

*Cerura vinula* (Linnaeus, 1758), also known as the Puss Moth ( Waring *et al*., 2017), is a moth in the family Notodontidae (now placed in subfamily Cerurinae, St Laurent *et al*., 2023) with around 29–38 mm wingspan ( Waring *et al*., 2017). This large moth is quite unmistakeable with its whitish or grey background colour, furry thorax, abdomen and forelegs, with black spots on thorax and base of forewings, and on the forewings a complex pattern of dark grey brackets making way for chevrons that increase in size towards the forewing termen and are interdigitated with dark lines radiating back from this edge. The female is larger and darker on average ( Waring *et al*., 2017). The antennae of the male are strongly pectinate. The moth displays thanatosis when disturbed, curling round its abdomen in a pseudo-stinging motion and displaying its yellow intersegmental membranes.

The adult moth flies in the United Kingdom from early April protracted to the end of July with a peak around the end of May, but emergence has advanced since the 1970s ( Randle *et al*., 2019: 226). The species is nocturnal and attracted to light.

In the UK the species is associated with a wide range of sallows, willows ( *Salix* L.) poplars and aspen ( *Populus* L.) in a wide variety of habitats including woods and gardens ( Randle *et al*., 2019); see Lepiforum (2025) for a list of foodplants.

The dark red, plant gall-like eggs of the Puss Moth are laid singly, in pairs or triplets on the upper leaf surface. The full-grown larva is spectacular, ventrally green with dorsal greyish-purple colouration that extends towards the second pair of prolegs like a saddle (see e.g. Lepiforum, 2025). On disturbance it displays a pair of red filament-like whips from its black and white striped caudal projections (which are modified anal prolegs) and the red area around the false thoracic face ( St Laurent *et al*., 2023) that is topped with two black eyespots and exposed in a prominent, aposematic display; the true legs are also black and white ringed. If attacked the larva can even project formic acid from the prothoracic cervical gland with a slit-like opening that is found in many notodontid moth caterpillars just under the head. Poulton (1890) discovered that this fluid contained 33–40% formic acid and he had himself felt the pain of droplets directed towards his eye. The early instar larva is mostly black with a pair of projections on the head and relatively long caudal projections. The adult larva becomes plum coloured when seeking a pupation site. The dark brown pupa is in an overwintering cocoon that is dome-shaped, hard as concrete, with woodchips included and well camouflaged on trunks of its foodplant or other substrates such as posts ( Lepiforum, 2025). The cocoon is so hard that only Greater Spotted Woodpeckers *Dendrocopos major* (Linnaeus, 1758) seem to be able to break them open ( Leverton & Cubitt, 2024). On emergence the adult utilises a secretion of potassium hydroxide to soften its exit ( Latter, 1895).

There are currently 16 512 records on the NBN Atlas Partnership (2025). The species occurs throughout Great Britain and Ireland, including the Hebrides, Scilly Islands, Channel Islands (Jersey and Guernsey), and Orkney, but not Shetland. However, records from Ireland are limited (see Randle *et al*., 2019 for details). The species is less frequent at higher elevations and northern latitudes ( Leverton & Cubitt, 2024; Randle *et al*., 2019; Waring *et al*., 2017). In the United Kingdom, distribution increased by 7% over 1970 to 2016, but declined by 13% from 2000 to 2016 ( Randle *et al*., 2019). The species has declined particularly in eastern Scotland, perhaps due to increased cocoon predation by Greater Spotted Woodpeckers ( Leverton & Cubitt, 2024). The species’ global range extends from the British and Irish Isles through Western Europe to Central Asia and Japan ( GBIF Secretariat, 2025).

The DNA barcode from the mitogenome assembly (OZ194647.1) is identical or very similar to a group of European haplotypes on BOLD (01/08/2025) sequenced from United Kingdom, Germany and Austria. It represents the COI-5P cluster (BIN) BOLD:AAB7277 with up to 3.37% variability ( *n* = 149), and thus exhibits a complex phylogeographic structure. This sequence falls into one of the two main haplogroups within this BIN, which essentially are identified as the nominotypical subspecies *C. vinula vinula* or *C. v. estonica* (Huene, 1905) at more northern latitudes, and another comprising taxa identified as not only the nominotypical subspecies but also ones identified as *C. vinula irakana* (Heydemann, Schulte & Remane, 1963), *C. intermedi*a* (Teich, 1896), *C. iberica* (Ortiz & Templado, 1966), *C. przewalskii* (Alphéraky, 1882), and *C. zolutuhini* Schintlmeister, 2008. The BIN also includes *C. felina* Butler, 1877, *C. hreblayi* Schintlmeister, 2008, *C. kautti* Schintlmeister, 2008, *C. himalayana* Moore, 1888 and *C. delavoiei* (Gaschet, 1876). The nearest neighbour to the BIN BOLD:AAB7277 at an average 1.59% *p*-distance is BOLD:AEI8479 (also identified as *C. delavoiei* (Gaschet, 1876)).

**Figure 1.**
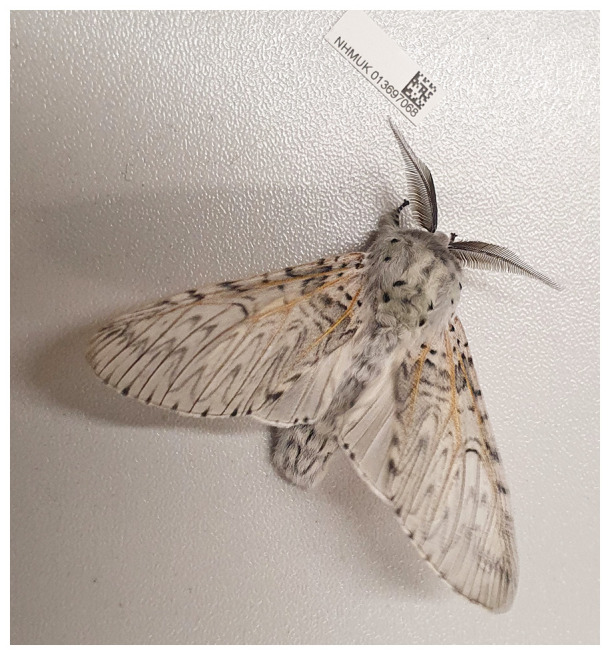
Photograph of the *Cerura vinula* (ilCerVinu1) specimen used for genome sequencing.

The genome will be helpful in investigating the phylogeography of the species in Europe and revising species and subspecies concepts. A sister relationship of ( *Cerura* Schrank, 1802 + *Kamalia* Koçak & Kemal, 2006) + *Furcula* Lamarck, 1816 was recovered using 28SrRNA by Kobayashi and Nonaka (2016). Systematics of the subfamily Cerurinae have recently been revised by St Laurent *et al*. (2023) based on data for 666 loci. *Cerura* is now one of 15 genera within the monophyletic subfamily Cerurinae. The shallowly diverged *Cerura vinula* complex, which included several of the above exemplar taxa, was recovered by St Laurent *et al*. (2023) as sister to the also European *Cerura erminea* (Esper, 1783) + the Asiatic *C. menciana* Moore, 1877, *Cerura* again being sister to the diverse Asian genus *Kamalia* and these two genera now being sister to the New World *Americerura* St Laurent and Goldstein, 2023. This whole clade was recovered as sister to the Indomalayan species *Neocerura liturata* (Walker, 1855) and all these taxa sister to *Neoharpyia* Daniel, 1965 + the Old World kitten moths (the genus *Furcula*). We present a chromosome-level genome sequence for *Cerura vinula*, the Puss Moth. The assembly was produced using the Tree of Life pipeline from a specimen collected in Lucas Road, High Wycombe, England, United Kingdom ( Figure 1), as part of the Darwin Tree of Life project.

## Methods

### Sample acquisition and DNA barcoding

The specimen used for genome sequencing was an adult male *Cerura vinula* (specimen ID NHMUK013697068, ToLID ilCerVinu1; Figure 1), collected from Lucas Road, High Wycombe, England, United Kingdom (latitude 51.63, longitude –0.74) on 2022-04-15. The specimen was collected and identified by David Lees (Natural History Museum). The same specimen was used for RNA sequencing. Sample metadata were collected in line with the Darwin Tree of Life project standards described by Lawniczak *et al*. (2022).

The initial identification was verified by an additional DNA barcoding process according to the framework developed by Twyford *et al.* (2024). A small sample was dissected from the specimen and stored in ethanol, while the remaining parts were shipped on dry ice to the Wellcome Sanger Institute (WSI) (see the protocol). The tissue was lysed, the COI marker region was amplified by PCR, and amplicons were sequenced and compared to the BOLD database, confirming the species identification ( Crowley *et al*., 2023). Following whole genome sequence generation, the relevant DNA barcode region was also used alongside the initial barcoding data for sample tracking at the WSI ( Twyford *et al*., 2024). The standard operating procedures for Darwin Tree of Life barcoding are available on protocols.io.

### Nucleic acid extraction

Protocols for high molecular weight (HMW) DNA extraction developed at the Wellcome Sanger Institute (WSI) Tree of Life Core Laboratory are available on protocols.io ( Howard *et al*., 2025). The ilCerVinu1 sample was weighed and triaged to determine the appropriate extraction protocol. Tissue from the head and thorax was homogenised by powermashing using a PowerMasher II tissue disruptor.

HMW DNA was extracted in the WSI Scientific Operations core using the Automated MagAttract v2 protocol. DNA was sheared into an average fragment size of 12–20 kb following the Megaruptor®3 for LI PacBio protocol. Sheared DNA was purified by automated SPRI (solid-phase reversible immobilisation). The concentration of the sheared and purified DNA was assessed using a Nanodrop spectrophotometer and Qubit Fluorometer using the Qubit dsDNA High Sensitivity Assay kit. Fragment size distribution was evaluated by running the sample on the FemtoPulse system. For this sample, the final post-shearing DNA had a Qubit concentration of 76.22 ng/μL and a yield of 3 582.34 ng, with a fragment size of 16.5 kb. The 260/280 spectrophotometric ratio was 1.8, and the 260/230 ratio was 1.53.

RNA was extracted from head and thorax tissue of ilCerVinu1 in the Tree of Life Laboratory at the WSI using the RNA Extraction: Automated MagMax™ *mir*Vana protocol. The RNA concentration was assessed using a Nanodrop spectrophotometer and a Qubit Fluorometer using the Qubit RNA Broad-Range Assay kit. Analysis of the integrity of the RNA was done using the Agilent RNA 6000 Pico Kit and Eukaryotic Total RNA assay.

### PacBio HiFi library preparation and sequencing

Library preparation and sequencing were performed at the WSI Scientific Operations core. Libraries were prepared using the SMRTbell Prep Kit 3.0 (Pacific Biosciences, California, USA), following the manufacturer’s instructions. The kit includes reagents for end repair/A-tailing, adapter ligation, post-ligation SMRTbell bead clean-up, and nuclease treatment. Size selection and clean-up were performed using diluted AMPure PB beads (Pacific Biosciences). DNA concentration was quantified using a Qubit Fluorometer v4.0 (ThermoFisher Scientific) and the Qubit 1X dsDNA HS assay kit. Final library fragment size was assessed with the Agilent Femto Pulse Automated Pulsed Field CE Instrument (Agilent Technologies) using the gDNA 55 kb BAC analysis kit.

The sample was sequenced on a Revio instrument (Pacific Biosciences). The prepared library was normalised to 2 nM, and 15 μL was used for making complexes. Primers were annealed and polymerases bound to generate circularised complexes, following the manufacturer’s instructions. Complexes were purified using 1.2X SMRTbell beads, then diluted to the Revio loading concentration (200–300 pM) and spiked with a Revio sequencing internal control. The sample was sequenced on a Revio 25M SMRT cell. The SMRT Link software (Pacific Biosciences), a web-based workflow manager, was used to configure and monitor the run and to carry out primary and secondary data analysis.

### Hi-C

***Sample preparation and crosslinking***

The Hi-C sample was prepared from 20–50 mg of frozen head and thorax tissue of the ilCerVinu1 sample using the Arima-HiC v2 kit (Arima Genomics). Following the manufacturer’s instructions, tissue was fixed and DNA crosslinked using TC buffer to a final formaldehyde concentration of 2%. The tissue was homogenised using the Diagnocine Power Masher-II. Crosslinked DNA was digested with a restriction enzyme master mix, biotinylated, and ligated. Clean-up was performed with SPRISelect beads before library preparation. DNA concentration was measured with the Qubit Fluorometer (Thermo Fisher Scientific) and Qubit HS Assay Kit. The biotinylation percentage was estimated using the Arima-HiC v2 QC beads.

***Hi-C library preparation and sequencing***

Biotinylated DNA constructs were fragmented using a Covaris E220 sonicator and size selected to 400–600 bp using SPRISelect beads. DNA was enriched with Arima-HiC v2 kit Enrichment beads. End repair, A-tailing, and adapter ligation were carried out with the NEBNext Ultra II DNA Library Prep Kit (New England Biolabs), following a modified protocol where library preparation occurs while DNA remains bound to the Enrichment beads. Library amplification was performed using KAPA HiFi HotStart mix and a custom Unique Dual Index (UDI) barcode set (Integrated DNA Technologies). Depending on sample concentration and biotinylation percentage determined at the crosslinking stage, libraries were amplified with 10–16 PCR cycles. Post-PCR clean-up was performed with SPRISelect beads. Libraries were quantified using the AccuClear Ultra High Sensitivity dsDNA Standards Assay Kit (Biotium) and a FLUOstar Omega plate reader (BMG Labtech).

Prior to sequencing, libraries were normalised to 10 ng/μL. Normalised libraries were quantified again and equimolar and/or weighted 2.8 nM pools. Pool concentrations were checked using the Agilent 4200 TapeStation (Agilent) with High Sensitivity D500 reagents before sequencing. Sequencing was performed using paired-end 150 bp reads on the Illumina NovaSeq X.

### RNA library preparation and sequencing

Libraries were prepared using the NEBNext ^®^ Ultra™ II Directional RNA Library Prep Kit for Illumina (New England Biolabs), following the manufacturer’s instructions. Poly(A) mRNA in the total RNA solution was isolated using oligo(dT) beads, converted to cDNA, and uniquely indexed; 14 PCR cycles were performed. Libraries were size-selected to produce fragments between 100–300 bp. Libraries were quantified, normalised, pooled to a final concentration of 2.8 nM, and diluted to 150 pM for loading. Sequencing was carried out on the Illumina NovaSeq X to generate 150-bp paired-end reads.

### Genome assembly

Prior to assembly of the PacBio HiFi reads, a database of *k*-mer counts ( *k* = 31) was generated from the filtered reads using FastK. GenomeScope2 ( Ranallo-Benavidez *et al*., 2020) was used to analyse the *k*-mer frequency distributions, providing estimates of genome size, heterozygosity, and repeat content.

The HiFi reads were assembled using Hifiasm in Hi-C phasing mode ( Cheng *et al*., 2021; Cheng *et al*., 2022), producing two haplotypes. Hi-C reads ( Rao *et al*., 2014) were mapped to the primary contigs using bwa-mem2 ( Vasimuddin *et al*., 2019). Contigs were further scaffolded with Hi-C data in YaHS ( Zhou *et al*., 2023), using the --break option for handling potential misassemblies. The scaffolded assemblies were evaluated using Gfastats ( Formenti *et al*., 2022), BUSCO ( Manni *et al*., 2021) and MERQURY.FK ( Rhie *et al*., 2020).

The mitochondrial genome was assembled using MitoHiFi ( Uliano-Silva *et al*., 2023), which runs MitoFinder ( Allio *et al*., 2020) and uses these annotations to select the final mitochondrial contig and to ensure the general quality of the sequence.

### Assembly curation

The assembly was decontaminated using the Assembly Screen for Cobionts and Contaminants ( ASCC) pipeline. TreeVal was used to generate the flat files and maps for use in curation. Manual curation was conducted primarily in PretextView and HiGlass ( Kerpedjiev *et al*., 2018). Scaffolds were visually inspected and corrected as described by Howe *et al*. (2021). Manual corrections included 8 breaks and 66 joins. The curation process is documented at https://gitlab.com/wtsi-grit/rapid-curation. PretextSnapshot was used to generate a Hi-C contact map of the final assembly.

### Assembly quality assessment

The Merqury.FK tool ( Rhie *et al*., 2020) was run in a Singularity container ( Kurtzer *et al*., 2017) to evaluate *k*-mer completeness and assembly quality for both haplotypes using the *k*-mer databases ( *k* = 31) computed prior to genome assembly. The analysis outputs included assembly QV scores and completeness statistics.

The genome was analysed using the BlobToolKit pipeline, a Nextflow implementation of the earlier Snakemake version ( Challis *et al*., 2020). The pipeline aligns PacBio reads using minimap2 ( Li, 2018) and SAMtools ( Danecek *et al*., 2021) to generate coverage tracks. It runs BUSCO ( Manni *et al*., 2021) using lineages identified from the NCBI Taxonomy ( Schoch *et al*., 2020). For the three domain-level lineages, BUSCO genes are aligned to the UniProt Reference Proteomes database ( Bateman *et al*., 2023) using DIAMOND blastp ( Buchfink *et al*., 2021). The genome is divided into chunks based on the density of BUSCO genes from the closest taxonomic lineage, and each chunk is aligned to the UniProt Reference Proteomes database with DIAMOND blastx. Sequences without hits are chunked using seqtk and aligned to the NT database with blastn ( Altschul *et al*., 1990). The BlobToolKit suite consolidates all outputs into a blobdir for visualisation. The BlobToolKit pipeline was developed using nf-core tooling ( Ewels *et al*., 2020) and MultiQC ( Ewels *et al*., 2016), with containerisation through Docker ( Merkel, 2014) and Singularity ( Kurtzer *et al*., 2017).

## Genome sequence report

### Sequence data

**Figure 2.**
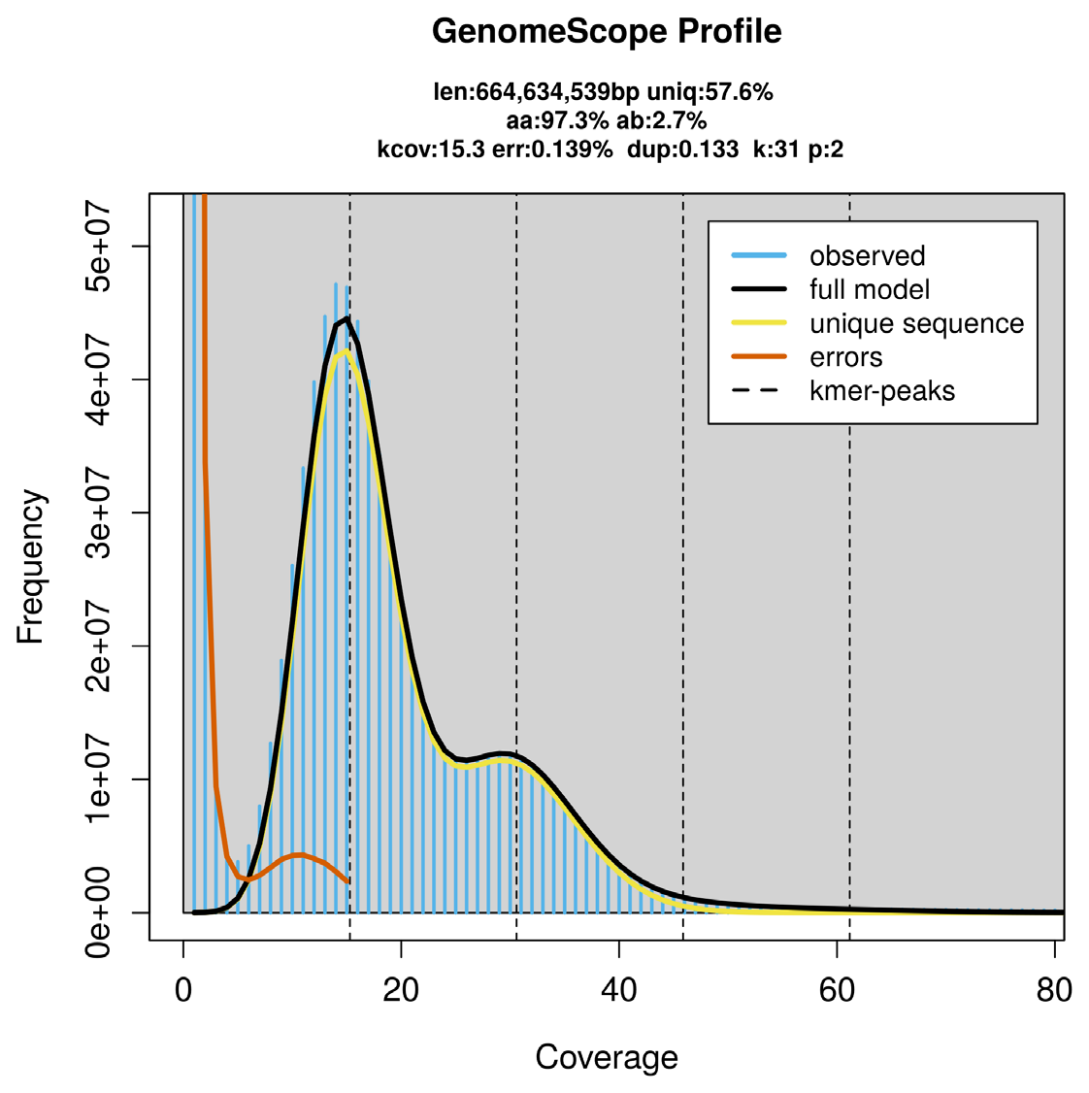
Frequency distribution of *k*-mers generated using GenomeScope2. The plot shows observed and modelled *k*-mer spectra, providing estimates of genome size, heterozygosity, and repeat content based on unassembled sequencing reads.

PacBio sequencing of the *Cerura vinula* specimen generated 21.28 Gb (gigabases) from 1.89 million reads, which were used to assemble the genome. GenomeScope2.0 analysis estimated the haploid genome size at 664.63 Mb, with a heterozygosity of 2.70% and repeat content of 42.69% ( Figure 2). These estimates guided expectations for the assembly. Based on the estimated genome size, the sequencing data provided approximately 31× coverage. Hi-C sequencing produced 105.35 Gb from 697.67 million reads, which were used to scaffold the assembly. RNA sequencing data were also generated and are available in public sequence repositories. Table 1 summarises the specimen and sequencing details.

**Table 1.** Specimen and sequencing data for BioProject PRJEB78778.

Platform	PacBio HiFi	Hi-C	RNA-seq
**ToLID**	ilCerVinu1	ilCerVinu1	ilCerVinu1
**Specimen ID**	NHMUK013697068	NHMUK013697068	NHMUK013697068
**BioSample (source individual)**	SAMEA114805616	SAMEA114805616	SAMEA114805616
**BioSample (tissue)**	SAMEA114805735	SAMEA114805735	SAMEA114805735
**Tissue**	head and thorax	head and thorax	head and thorax
**Instrument**	Revio	Illumina NovaSeq X	Illumina NovaSeq X
**Run accessions**	ERR13485735	ERR13493992	ERR14792842
**Read count total**	1.89 million	697.67 million	92.11 million
**Base count total**	21.28 Gb	105.35 Gb	13.91 Gb

### Assembly statistics

The genome was assembled into two haplotypes using Hi-C phasing. Haplotype 1 was curated to chromosome level, while haplotype 2 was assembled to scaffold level. The final assembly has a total length of 690.59 Mb in 69 scaffolds, with 298 gaps, and a scaffold N50 of 39.49 Mb ( Table 2).

**Table 2.** Genome assembly statistics.

**Assembly name**	ilCerVinu1.hap1.1	ilCerVinu1.hap2.1
**Assembly accession**	GCA_964276795.1	GCA_964276825.1
**Assembly level**	chromosome	scaffold
**Span (Mb)**	690.59	688.27
**Number of chromosomes**	19	N/A
**Number of contigs**	367	328
**Contig N50**	3.74 Mb	3.89 Mb
**Number of scaffolds**	69	59
**Scaffold N50**	39.49 Mb	39.6 Mb
**Longest scaffold length (Mb)**	49.71	N/A
**Sex chromosomes**	Z	N/A
**Organelles**	Mitochondrion: 16.79 kb	N/A

**Figure 3.**
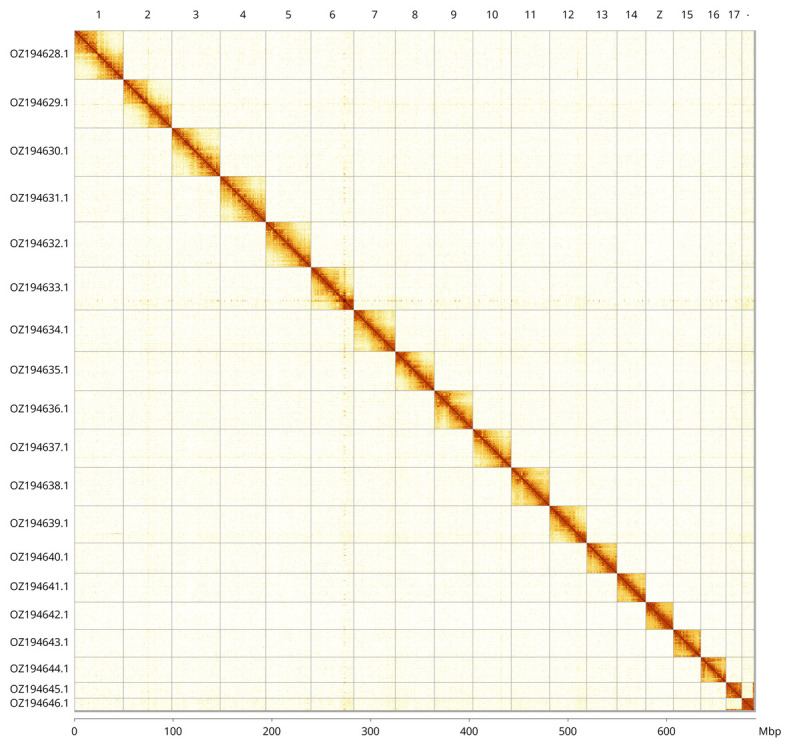
Hi-C contact map of the *Cerura vinula* genome assembly. Assembled chromosomes are shown in order of size and labelled along the axes, with a megabase scale shown below. The plot was generated using PretextSnapshot.

Most of the assembly sequence (99.72%) was assigned to 19 chromosomal-level scaffolds, representing 18 autosomes and the Z sex chromosome. These chromosome-level scaffolds, confirmed by Hi-C data, are named according to size ( Figure 3; Table 3). The Z chromosome was identified through synteny analysis with the genome of *Clostera curtula* (GCA_905475355.2). During curation we noted that contigs in the following regions have uncertain order and orientation: chromosome 6 (33.2–34.9 Mbp) and chromosome 18 (11.2–12.39 Mbp).

**Table 3.** Chromosomal pseudomolecules in the haplotype 1 genome assembly of *Cerura vinula* ilCerVinu1.

INSDC accession	Molecule	Length (Mb)	GC%
OZ194628.1	1	49.71	38
OZ194629.1	2	49.03	38.50
OZ194630.1	3	48.96	38.50
OZ194631.1	4	46.23	38.50
OZ194632.1	5	45.98	38
OZ194633.1	6	43.23	39
OZ194634.1	7	42.14	38.50
OZ194635.1	8	39.49	38.50
OZ194636.1	9	39.05	38.50
OZ194637.1	10	38.94	38.50
OZ194638.1	11	38.85	38.50
OZ194639.1	12	37.43	38.50
OZ194640.1	13	30.74	38
OZ194641.1	14	29.19	38
OZ194643.1	15	27.90	38
OZ194644.1	16	25.43	38
OZ194645.1	17	16.01	39
OZ194646.1	18	12.42	40.50
OZ194642.1	Z	27.94	37

The mitochondrial genome was also assembled. This sequence is included as a contig in the multifasta file of the genome submission and as a standalone record.

**Figure 4.**
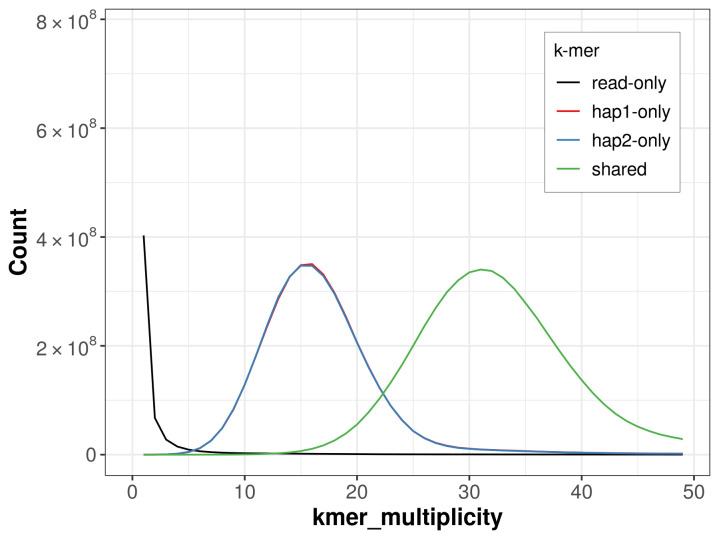
Evaluation of *k*-mer completeness using MerquryFK. This plot illustrates the recovery of *k*‐mers from the original read data in the final assemblies. The horizontal axis represents *k*‐mer multiplicity, and the vertical axis shows the number of *k*‐mers. The black curve represents *k*‐mers that appear in the reads but are not assembled. The green curve corresponds to *k*‐mers shared by both haplotypes, and the red and blue curves show *k*‐mers found only in one of the haplotypes.

For haplotype 1, the estimated QV is 64.1, and for haplotype 2, 64.1. When the two haplotypes are combined, the assembly achieves an estimated QV of 64.1. The *k*-mer completeness is 63.93% for haplotype 1, 63.88% for haplotype 2, and 99.36% for the combined haplotypes ( Figure 4).

**Figure 5.**
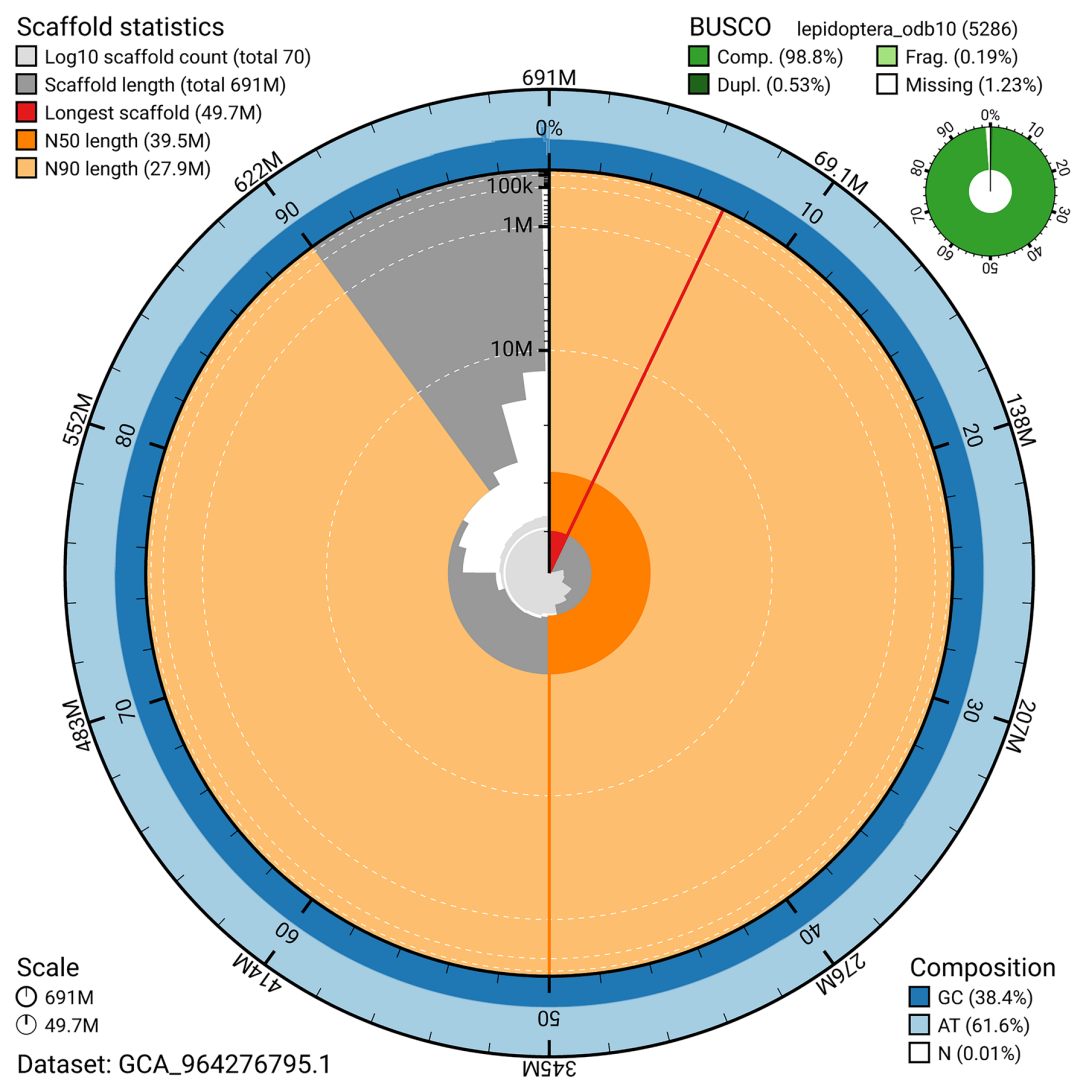
Assembly metrics for ilCerVinu1.hap1.1. The BlobToolKit snail plot provides an overview of assembly metrics and BUSCO gene completeness. The circumference represents the length of the whole genome sequence, and the main plot is divided into 1 000 bins around the circumference. The outermost blue tracks display the distribution of GC, AT, and N percentages across the bins. Scaffolds are arranged clockwise from longest to shortest and are depicted in dark grey. The longest scaffold is indicated by the red arc, and the deeper orange and pale orange arcs represent the N50 and N90 lengths. A light grey spiral at the centre shows the cumulative scaffold count on a logarithmic scale. A summary of complete, fragmented, duplicated, and missing BUSCO genes in the set is presented at the top right. An interactive version of this figure can be accessed on the BlobToolKit viewer.

**Figure 6.**
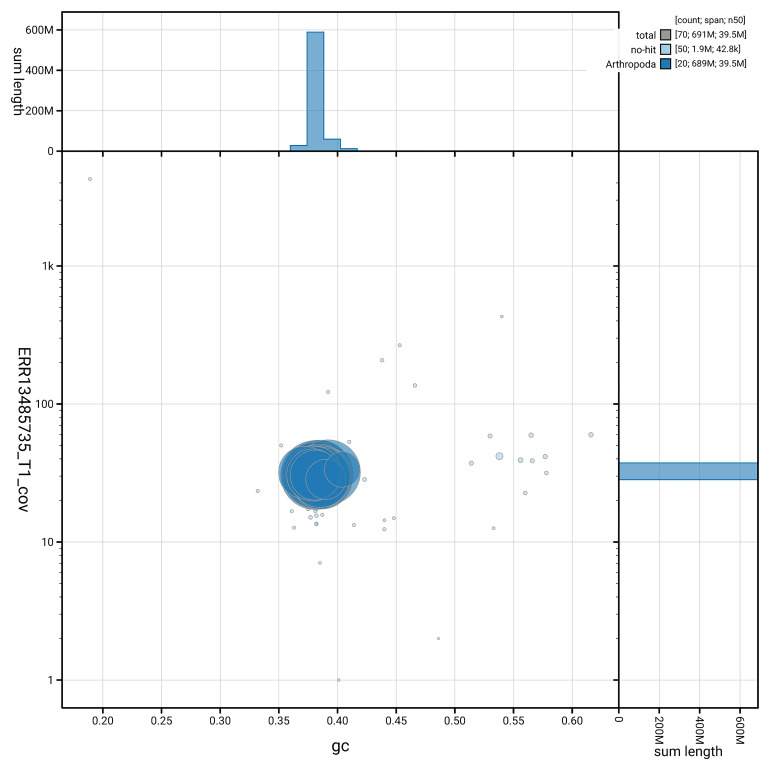
BlobToolKit GC-coverage plot for ilCerVinu1.hap1.1. Blob plot showing sequence coverage (vertical axis) and GC content (horizontal axis). The circles represent scaffolds, with the size proportional to scaffold length and the colour representing phylum membership. The histograms along the axes display the total length of sequences distributed across different levels of coverage and GC content. An interactive version of this figure is available on the BlobToolKit viewer.

BUSCO analysis using the lepidoptera_odb10 reference set ( *n* = 5 286) identified 98.8% of the expected gene set (single = 98.2%, duplicated = 0.5%) for haplotype 1. The snail plot in Figure 5 summarises the scaffold length distribution and other assembly statistics for haplotype 1. The blob plot in Figure 6 shows the distribution of scaffolds by GC proportion and coverage for haplotype 1.

Table 4 lists the assembly metric benchmarks adapted from Rhie *et al.* (2021) the Earth BioGenome Project Report on Assembly Standards September 2024. The EBP metric, calculated for the haplotype 1, is **6.C.Q64**, meeting the recommended reference standard.

**Table 4.** Earth Biogenome Project summary metrics for the *Cerura vinula* assembly.

Measure	Value	Benchmark
EBP summary (haplotype 1)	6.C.Q64	6.C.Q40
Contig N50 length	3.74 Mb	≥ 1 Mb
Scaffold N50 length	39.49 Mb	= chromosome N50
Consensus quality (QV)	Haplotype 1: 64.1; haplotype 2: 64.1; combined: 64.1	≥ 40
*k*-mer completeness	Haplotype 1: 63.93%; Haplotype 2: 63.88%; combined: 99.36%	≥ 95%
BUSCO	C:98.8% [S:98.2%; D:0.5%]; F:0.2%; M:1.0%; n:5 286	S > 90%; D < 5%
Percentage of assembly assigned to chromosomes	99.72%	≥ 90%

### Wellcome Sanger Institute – Legal and Governance

The materials that have contributed to this genome note have been supplied by a Darwin Tree of Life Partner. The submission of materials by a Darwin Tree of Life Partner is subject to the **‘Darwin Tree of Life Project Sampling Code of Practice’**, which can be found in full on the Darwin Tree of Life website. By agreeing with and signing up to the Sampling Code of Practice, the Darwin Tree of Life Partner agrees they will meet the legal and ethical requirements and standards set out within this document in respect of all samples acquired for, and supplied to, the Darwin Tree of Life Project. Further, the Wellcome Sanger Institute employs a process whereby due diligence is carried out proportionate to the nature of the materials themselves, and the circumstances under which they have been/are to be collected and provided for use. The purpose of this is to address and mitigate any potential legal and/or ethical implications of receipt and use of the materials as part of the research project, and to ensure that in doing so we align with best practice wherever possible. The overarching areas of consideration are:

- Ethical review of provenance and sourcing of the material
- Legality of collection, transfer and use (national and international)

Each transfer of samples is further undertaken according to a Research Collaboration Agreement or Material Transfer Agreement entered into by the Darwin Tree of Life Partner, Genome Research Limited (operating as the Wellcome Sanger Institute), and in some circumstances, other Darwin Tree of Life collaborators.

## Data Availability

European Nucleotide Archive: Cerura vinula. Accession number PRJEB78778. The genome sequence is released openly for reuse. The *Cerura vinula* genome sequencing initiative is part of the Darwin Tree of Life Project (PRJEB40665), the Sanger Institute Tree of Life Programme (PRJEB43745) and Project Psyche (PRJEB71705). All raw sequence data and the assembly have been deposited in INSDC databases. The genome will be annotated using available RNA-Seq data and presented through the Ensembl pipeline at the European Bioinformatics Institute. Raw data and assembly accession identifiers are reported in Table 1 and Table 2. Production code used in genome assembly at the WSI Tree of Life is available at https://github.com/sanger-tol. Table 5 lists software versions used in this study.

## Author information

Contributors are listed at the following links:

- Members of the University of Oxford and Wytham Woods Genome Acquisition Lab
- Members of the Natural History Museum Genome Acquisition Lab
- Members of the Marine Biological Association Genome Acquisition Lab
- Members of the Darwin Tree of Life Barcoding collective
- Members of the Wellcome Sanger Institute Tree of Life Management, Samples and Laboratory team
- Members of Wellcome Sanger Institute Scientific Operations – Sequencing Operations
- Members of the Wellcome Sanger Institute Tree of Life Core Informatics team
- Members of the Tree of Life Core Informatics collective
- Members of the Darwin Tree of Life Consortium

## References

[ref-1] Allio R Schomaker-Bastos A Romiguier J : MitoFinder: efficient automated large-scale extraction of mitogenomic data in target enrichment phylogenomics. *Mol Ecol Resour.* 2020;20(4):892–905. 10.1111/1755-0998.13160 32243090 PMC7497042

[ref-2] Altschul SF Gish W Miller W : Basic Local Alignment Search Tool. *J Mol Biol.* 1990;215(3):403–410. 10.1016/S0022-2836(05)80360-2 2231712

[ref-3] Bateman A Martin MJ Orchard S : UniProt: the Universal Protein Knowledgebase in 2023. *Nucleic Acids Res.* 2023;51(D1):D523–D531. 10.1093/nar/gkac1052 36408920 PMC9825514

[ref-4] Buchfink B Reuter K Drost HG : Sensitive protein alignments at Tree-of-Life scale using DIAMOND. *Nat Methods.* 2021;18(4):366–368. 10.1038/s41592-021-01101-x 33828273 PMC8026399

[ref-5] Challis R Richards E Rajan J : BlobToolKit – interactive quality assessment of genome assemblies. *G3 (Bethesda).* 2020;10(4):1361–1374. 10.1534/g3.119.400908 32071071 PMC7144090

[ref-6] Cheng H Concepcion GT Feng X : Haplotype-resolved *de novo* assembly using phased assembly graphs with hifiasm. *Nat Methods.* 2021;18(2):170–175. 10.1038/s41592-020-01056-5 33526886 PMC7961889

[ref-7] Cheng H Jarvis ED Fedrigo O : Haplotype-resolved assembly of diploid genomes without parental data. *Nat Biotechnol.* 2022;40(9):1332–1335. 10.1038/s41587-022-01261-x 35332338 PMC9464699

[ref-8] Crowley L Allen H Barnes I : A sampling strategy for genome sequencing the British terrestrial Arthropod fauna [version 1; peer review: 2 approved]. *Wellcome Open Res.* 2023;8:123. 10.12688/wellcomeopenres.18925.1 37408610 PMC10318377

[ref-9] Danecek P Bonfield JK Liddle J : Twelve years of SAMtools and BCFtools. *GigaScience.* 2021;10(2): giab008. 10.1093/gigascience/giab008 33590861 PMC7931819

[ref-10] Ewels P Magnusson M Lundin S : MultiQC: summarize analysis results for multiple tools and samples in a single report. *Bioinformatics.* 2016;32(19):3047–3048. 10.1093/bioinformatics/btw354 27312411 PMC5039924

[ref-11] Ewels PA Peltzer A Fillinger S : The nf-core framework for community-curated bioinformatics pipelines. *Nat Biotechnol.* 2020;38(3):276–278. 10.1038/s41587-020-0439-x 32055031

[ref-12] Formenti G Abueg L Brajuka A : Gfastats: conversion, evaluation and manipulation of genome sequences using assembly graphs. *Bioinformatics.* 2022;38(17):4214–4216. 10.1093/bioinformatics/btac460 35799367 PMC9438950

[ref-13] GBIF Secretariat: *Cerura vinula* Linnaeus, 1758.2025. Reference Source

[ref-14] Howard C Denton A Jackson B : On the path to reference genomes for all biodiversity: lessons learned and laboratory protocols created in the Sanger Tree of Life core laboratory over the first 2000 species. *bioRxiv.* 2025. 10.1101/2025.04.11.648334 PMC12548527 41129326

[ref-15] Howe K Chow W Collins J : Significantly improving the quality of genome assemblies through curation. *GigaScience.* 2021;10(1): giaa153. 10.1093/gigascience/giaa153 33420778 PMC7794651

[ref-16] Kerpedjiev P Abdennur N Lekschas F : HiGlass: web-based visual exploration and analysis of genome interaction maps. *Genome Biol.* 2018;19(1): 125. 10.1186/s13059-018-1486-1 30143029 PMC6109259

[ref-17] Kobayashi H Nonaka M : Molecular phylogeny of the Notodontidae: subfamilies inferred from 28S rRNA sequences (Lepidoptera, Noctuoidea, Notodontidae). *Tinea.* 2016;23:1–83. Reference Source

[ref-18] Kurtzer GM Sochat V Bauer MW : Singularity: scientific containers for mobility of compute. *PLoS One.* 2017;12(5): e0177459. 10.1371/journal.pone.0177459 28494014 PMC5426675

[ref-19] Latter OH : XIV. Further notes on the secretion of potassium hydroxide by *Dicranura vinula* (imago), and similar phenomena in other Lepidoptera. *Transactions of the Entomological Society of London.* 1895;43:399–411.

[ref-20] Lawniczak MKN Davey RP Rajan J : Specimen and sample metadata standards for biodiversity genomics: a proposal from the Darwin Tree of Life project [version 1; peer review: 2 approved with reservations]. *Wellcome Open Res.* 2022;7:187. 10.12688/wellcomeopenres.17605.1

[ref-21] Lepiforum: *Cerura vinula* (Linnaeus, 1758) Großer Gabelschwanz. 2025. Reference Source

[ref-22] Leverton R Cubitt M : The larger moths of Scotland.Atropos Books,2024. Reference Source

[ref-23] Li H : Minimap2: pairwise alignment for nucleotide sequences. *Bioinformatics.* 2018;34(18):3094–3100. 10.1093/bioinformatics/bty191 29750242 PMC6137996

[ref-24] Manni M Berkeley MR Seppey M : BUSCO update: novel and streamlined workflows along with broader and deeper phylogenetic coverage for scoring of eukaryotic, prokaryotic, and viral genomes. *Mol Biol Evol.* 2021;38(10):4647–4654. 10.1093/molbev/msab199 34320186 PMC8476166

[ref-25] Merkel D : Docker: lightweight Linux containers for consistent development and deployment. *Linux J.* 2014;2014(239): 2. Reference Source

[ref-26] NBN Atlas Partnership: *Cerura vinula* (Linnaeus, 1758) Puss Moth. 2025. Reference Source 10.12688/wellcomeopenres.24919.1 PMC12789857 41523162

[ref-27] Poulton EB : The colours of animals.London: Kegan Paul, Trench, Trübner & Co Ltd,1890. Reference Source

[ref-28] Ranallo-Benavidez TR Jaron KS Schatz MC : GenomeScope 2.0 and Smudgeplot for reference-free profiling of polyploid genomes. *Nat Commun.* 2020;11(1): 1432. 10.1038/s41467-020-14998-3 32188846 PMC7080791

[ref-29] Randle Z Evans-Hill LJ Parsons MS : Atlas of Britain & Ireland’s larger moths.Newbury: Pisces Publications,2019. Reference Source

[ref-30] Rao SSP Huntley MH Durand NC : A 3D map of the human genome at kilobase resolution reveals principles of chromatin looping. *Cell.* 2014;159(7):1665–1680. 10.1016/j.cell.2014.11.021 25497547 PMC5635824

[ref-31] Rhie A McCarthy SA Fedrigo O : Towards complete and error-free genome assemblies of all vertebrate species. *Nature.* 2021;592(7856):737–746. 10.1038/s41586-021-03451-0 33911273 PMC8081667

[ref-32] Rhie A Walenz BP Koren S : Merqury: reference-free quality, completeness, and phasing assessment for genome assemblies. *Genome Biol.* 2020;21(1): 245. 10.1186/s13059-020-02134-9 32928274 PMC7488777

[ref-33] Schoch CL Ciufo S Domrachev M : NCBI Taxonomy: a comprehensive update on curation, resources and tools. *Database (Oxford).* 2020;2020: baaa062. 10.1093/database/baaa062 32761142 PMC7408187

[ref-34] St Laurent RA Goldstein PZ Miller JS : Phylogenetic systematics, diversification, and biogeography of cerurinae (lepidoptera: Notodontidae) and a description of a new genus. *Insect Syst Divers.* 2023;7(2):3. 10.1093/isd/ixad004

[ref-35] Twyford AD Beasley J Barnes I : A DNA barcoding framework for taxonomic verification in the Darwin Tree of Life project [version 1; peer review: 2 approved]. *Wellcome Open Res.* 2024;9:339. 10.12688/wellcomeopenres.21143.1 39386966 PMC11462125

[ref-36] Uliano-Silva M Ferreira JGRN Krasheninnikova K : MitoHiFi: a python pipeline for mitochondrial genome assembly from PacBio High Fidelity reads. *BMC Bioinformatics.* 2023;24(1): 288. 10.1186/s12859-023-05385-y 37464285 PMC10354987

[ref-37] Vasimuddin M Misra S Li H : Efficient architecture-aware acceleration of BWA-MEM for multicore systems.In: *2019 IEEE International Parallel and Distributed Processing Symposium (IPDPS).*IEEE,2019;314–324. 10.1109/IPDPS.2019.00041

[ref-38] Waring P Townsend M Lewington R : Field guide to the moths of Great Britain and Ireland.Bloomsbury Publishing,2017. Reference Source

[ref-39] Zhou C McCarthy SA Durbin R : YaHS: Yet another Hi-C Scaffolding tool. *Bioinformatics.* 2023;39(1): btac808. 10.1093/bioinformatics/btac808 36525368 PMC9848053

