# Supplementary material for: The genome sequence of the Puss Moth, Cerura vinula (Linnaeus, 1758) (Lepidoptera: Notodontidae)
Source: Wellcome Open Res. 2025 Oct 3;10:532. [Version 1] doi: 10.12688/wellcomeopenres.24919.1 (PMC12789857; doi:10.12688/wellcomeopenres.24919.1)
Supplement: Peer review history

# Reviewer report: Reviewer response for version 1

**DOI:** 10.21956/wellcomeopenres.27449.r135160

**Published:** 2026-01-09

**Authors:** Annabel Whibley

**Affiliations:** 1 Grapevine Improvement, Bragato Research Institute, Lincoln, New Zealand

Lees and colleagues report the genome assembly of the Puss Moth ( *Cerura vinula*). The background section provides a fascinating distillation of the natural history of the species and also carefully sets out the phylogenetic complexities in the taxon which have been reported by barcode sequence variation.

 The sequencing process, assembly and reporting follow standard DToL templates. Metadata and pipelines for assembly and evaluation are comprehensive and public accession links are active. RNAseq data has been generated but an annotation has not been reported. The genome assembly quality is excellent (formally 6.C.Q64).  The Genomescope2 plot indicates high heterozygosity (~2.7%) and in this context the reporting of two haplotypes is helpful. Haplotype 1 was curated to pseudochromosome level (with >99.7% of the assembly assigned to chromosomes), whilst Haplotype 2 is presented at scaffold level but looks, by assembly length and scaffold count, at least, to have good completeness and contiguity.

 Minor comments:

 I have noted in previous reviews that the omission of the RIN score and RNA yields when other nucleic acid metrics are provided seems odd. I also find the  templated “…Agilent RNA 6000 Pico Kit and Eukaryotic Total RNA assay” wording difficult- it makes the assay sound separate from the Pico Kit.

Are sufficient details of methods and materials provided to allow replication by others?

Yes

Is the rationale for creating the dataset(s) clearly described?

Yes

Are the datasets clearly presented in a useable and accessible format?

Yes

Are the protocols appropriate and is the work technically sound?

Yes

Reviewer Expertise:

Genomics, Bioinformatics, Evolution

I confirm that I have read this submission and believe that I have an appropriate level of expertise to confirm that it is of an acceptable scientific standard.

# Reviewer report: Reviewer response for version 1

**DOI:** 10.21956/wellcomeopenres.27449.r138051

**Published:** 2025-11-25

**Authors:** Panagiotis Ioannidis

**Affiliations:** 1 Foundation for Research & Technology - Hellas, Crete, Greece

This paper describes the sequencing and assembly of the lepidopteran Cerura vinula. The introductory information are very detailed and therefore give a very good overview of the ecology and biology of this species. Maybe the only thing missing is one (or more!) photo of the spectacular larva!

 The assembly is very good, which is typical for the assemblies generated from long reads + Hi-C data. More specifically, virtually the entire assembly is assigned to one of the 19 chromosomal-level scaffolds (CC ratio for hap1 = 69/19 = 3.63), QV is >60, k-mer completeness is >99% and BUSCO score is 98.8%. The blobplot (fig 6) shows that there are virtually no other scaffolds, other than the 19 expected ones.

 All in all, it's a very good assembly. The only thing missing is a gene set. I noticed that the authors mention that such a task will be carried out at Ensembl which is good and will greatly facilitate the use of this genome by a much broader number of scientists.

Are sufficient details of methods and materials provided to allow replication by others?

Yes

Is the rationale for creating the dataset(s) clearly described?

Yes

Are the datasets clearly presented in a useable and accessible format?

Yes

Are the protocols appropriate and is the work technically sound?

Yes

Reviewer Expertise:

insect genomics; bioinformatics

I confirm that I have read this submission and believe that I have an appropriate level of expertise to confirm that it is of an acceptable scientific standard.
